# Insidious Headache: Brain Toxoplasma Abscess

**DOI:** 10.1590/0037-8682-0212-2022

**Published:** 2022-08-12

**Authors:** Alptekin Tosun, Merve Nur Tasdemir, Emrah Sulun

**Affiliations:** 1 Giresun University, Faculty of Medicine, Department of Radiology, Giresun, Turkey.

**FIGURE 1:**
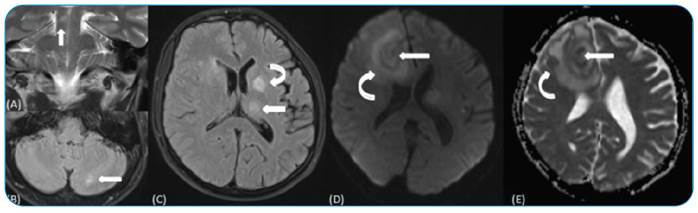
**(A)** Coronal T2-weighted image demonstrates an indistinct intense lesion on the cerebral peduncle (arrow); **(B)** axial FLAIR image shows similar focus on the left cerebellar hemisphere (arrow); **(C)** axial FLAIR image indicates thalamic (arrow) and putaminal (curved arrow) intense lesions; **(D-E)** and DWI and apparent diffusion coefficient map reveals restricted diffusion within the lesion and capsule (arrow), and extensive vasogenic edema (curved arrow). **DWI:** diffusion weighted imaging; **FLAIR:** fluid-attenuated inversion recovery.

**FIGURE 2:**
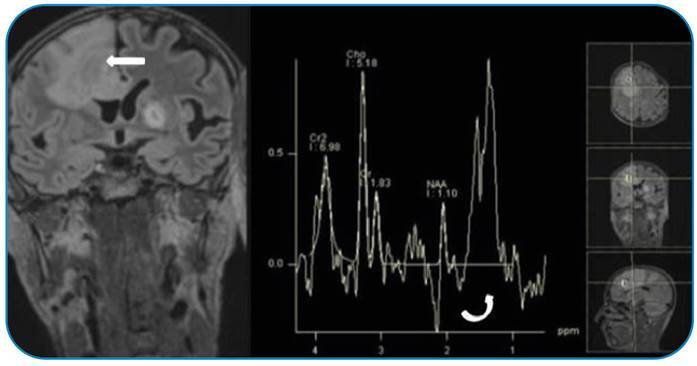
MR spectroscopy image of the right cerebral hemispheric lesion (arrow) exhibits increased choline/creatine and decreased NAA/creatine ratios by the existence of lipid-lactate peaks (curved arrow).

A 47-year-old man was admitted to the emergency department with headache, weight loss, and night sweats; “B symptoms” were investigated and confirmed by Western blot. Headache was considered a nonspecific symptom. On *magnetic resonance imaging (MRI)* in the thalamus, putamen, and cerebral peduncle, as well as the cerebral and cerebellar hemispheres, there were detected multiple cystic lesions. The majority of the lesions had central cystic components with thickened capsules. Restricted diffusion was noted in the lesion and capsule with extensive peripheral vasogenic edema on diffusion-weighted imaging (Figure 1). Magnetic resonance spectroscopy, that does not require the use of contrast material, revealed high choline and low N-acetyl aspartate metabolites coexisting with lactate and lipid peaks highlighted inflammatory conditions (Figure 2). The patient underwent surgical biopsy for diagnosis and therapeutic drainage. Histopathology findings are consistent with a nonspecific infection. Due to antitoxoplasma Immunoglobulin G positivity, the patient was treated with pyrimethamine 50 mg once a day and sulfadiazine 500 mg four times a day for six weeks. In the posttreatment control MRI, the size, number, and morphology of the lesions decreased, and the diagnosis was made retrospectively.

Toxoplasmosis is an opportunistic central nervous system infection^1^. The most common symptoms with cerebral toxoplasmosis are headaches, and fever^2^ ^,^ ^3^.

In our experience with this disease, insidious headaches with abnormal foci are present and presumably expound to an abscess. Although a brain abscess is not specific in the disease course, it is an important diagnostic consideration. Abnormal brain foci should be evaluated using advanced MRI techniques.
